# SARS-CoV-2 infection susceptibility influenced by *ACE2* genetic polymorphisms: insights from Tehran Cardio-Metabolic Genetic Study

**DOI:** 10.1038/s41598-020-80325-x

**Published:** 2021-01-15

**Authors:** Hossein Lanjanian, Maryam Moazzam-Jazi, Mehdi Hedayati, Mahdi Akbarzadeh, Kamran Guity, Bahareh Sedaghati-khayat, Fereidoun Azizi, Maryam S. Daneshpour

**Affiliations:** 1 Cellular and Molecular Endocrine Research Center, Research Institute for Endocrine Sciences, Shahid Beheshti University of Medical Sciences, POBox: 19195-4763, Tehran, Iran; 2 Endocrine Research Center, Research Institute for Endocrine Sciences, Shahid Beheshti University of Medical Sciences, Tehran, Iran

**Keywords:** Genetic variation, Personalized medicine, Molecular modelling

## Abstract

The genetic variations among individuals are one of the notable factors determining disease severity and drug response. Nowadays, COVID-19 pandemic has been adversely affecting many aspects of human life. We used the Tehran Cardio-Metabolic Genetic Study (TCGS) data that is an ongoing genetic study including the whole-genome sequencing of 1200 individuals and chip genotyping of more than 15,000 participants. Here, the effect of *ACE2* variations by focusing on the receptor-binding site of SARS-CoV-2 and ACE2 cleavage by TMPRSS2 protease were investigated through simulations study. After analyzing TCGS data, 570 genetic variations on the *ACE2* gene, including single nucleotide polymorphisms (SNP) and insertion/deletion (INDEL) were detected. Interestingly, two observed missense variants, K26R and S331F, which only the first one was previously reported, can reduce the receptor affinity for the viral Spike protein. Moreover, our bioinformatics simulation of 3D structures and docking of proteins explains important details of ACE2-Spike and ACE2-TMPRSS2 interactions, especially the critical role of Arg652 of ACE2 for protease function of TMPRSS2 was uncovered. As our results show that the genetic variation of *ACE2* can at least influence the affinity of this receptor to its partners, we need to consider the genetic variations on *ACE2* as well as other genes in the pathways that contribute to the pathogenesis of COVID-19 for designing efficient drugs and vaccines.

## Introduction

There are some historical pieces of evidence that ethnic and geographical differences play a role in the susceptibility to disease. Some studies considered the role of socioeconomic factors in explaining racial differences in health status. In addition to the social and cultural differences, infectious sources, and transmission routes, the genetic variations among individuals are also of the main factor in response to different diseases^1–3^.

The two most prominent features in the molecular biology of any diseases and treatments are genetic variations and gene expression. These factors are the hidden layers of biochemical signaling, cell proliferation, and metabolism in the alive creatures. On the other hand, pharmacogenomics as the growing field of research and development focuses on the effects of genetic variations on the response to drugs^4,5^. Therefore, to battle the present unknown enemy, it is better to investigate all molecular aspects of the virus pathogenesis.

Similar to SARS-CoV, the receptor-binding domain (RBD) of the SARS-CoV-2 Spike protein recognizes the angiotensin-converting enzyme 2 (ACE2) as a host receptor^6–10^. The host susceptibility to SARS-CoV mostly depends on the affinity between the host ACE2 and the viral RBD, e.g. the residues near lysine 31, tyrosine 41, 82–84, and 353–357 in human ACE2 were imperative for the binding of Spike protein in coronavirus^11,12^; Hence, any genetic variations at or near these above-mentioned positions have the potential to affect the receptor affinity to the virus and the corresponding viral infectivity. Additionally, the SARS-CoV infection is affected by the host cell proteases; the proteolytically processing of ACE2 following the virus attachment to the receptor may play a key role in SARS-CoV entrance and pathogenesis^8,13^. It has been reported that the activity of a member of the A Disintegrin And Metalloproteinase family (ADAM17) is required for the ACE2 cleavage and shedding into the extracellular space. It can facilitate the virus uptake to the host cells and promote the SARS pathogenesis. The positions of Arg708-Ser709 in human ACE2 were considered as the putative site for ADAM17-mediated cleavage^14^. Moreover, ADAM17 and Transmembrane Serine Protease 2 (TMPRSS2) compete for the ACE2 cleavage. ACE2 proteolysis through TMPRSS2 protease is the critical process to augment the SARS-S-mediated entry to the target cells^15^.

However, the negative correlation between the *ACE2* expression and SARS-CoV severity was reported; the *ACE2* transcript level is reduced at a higher dosage of SARS-CoV-2 or with time post-infection of virus^16^. Consequently, not only the SARS virus binding to the ACE2 receptor and subsequent infection but also the ACE2 depletion from the cell surface can lead to severe deterioration of lung tissues^17,18^. The higher risk of SARS-CoV-2 infection in patients with complex diseases like hypertension and diabetes might have resulted from the association of *ACE2* genetic variations with these diseases^19^.

In the current study, all of the *ACE2* genetic variations within Tehran Cardio-Metabolic Genetic Study (TCGS)^20^, representative of the Iranian population were investigated. To examine the functional effects of these variations on the corresponding interactions and considering that the ACE2 and TMPRSS2 interaction, as well as ACE2 and Spike protein interaction, have not been completely understood, we surveyed the three-dimensional structures as well as the binding sites of the ACE2 and TMPRSS2, B(0)AT1, and Spike protein to simulate the protein–protein interactions of wild type and mutated forms. Additionally, we examined the effect of key variations on the *ACE2* and *TMPRSS2* expression using publicly available data.

## Methods

### Sample selection

In this study, Iranian subjects were selected from the TCGS project that is a part of an ongoing Tehran Lipid and Glucose Study (TLGS) cohort^21^, which was designed in collaboration with the Research Institute for Endocrine Sciences (RIES) and DeCODE genetic company. TCGS participants have been genotyped and followed up for cardio-metabolic risk factors every 3 years since 1999 (1999-now)^20^. Moreover, 800 Iranian participants in the Iranome project^22^ were also utilized. Additionally, 2504 individuals from five populations of the 1000 Genome Project (African, American, East Asian, European, and South Asian)^23^ and China population^24^ were used.

### Genotype data

For the TCGS project, more than 15,000 individuals were included. Blood samples were washed with lysis buffer where PBS and RBCs were separated. Then, through the alkaline boiling method, DNA was extracted from the WBCs and the cell extracts were stored at – 20 °C. Quantitative and qualitative assessments on the extracted DNA were performed by electrophoresis and spectrophotometry. Genomic samples were genotyped by Human OmniExpress-24-v1-0 (Illumina Inc., San Diego, CA) chip. As well, the whole genome of 1162 TCGS participants at this project were sequenced using the Illumina HiSeq platform with the average coverage of 35X. Informed written consent had been obtained from all participants. The study was approved by the ethics committee of the Research Institute for Endocrine Sciences. In summary, after checking the read quality control, the raw reads mapped with the human reference genome assembly (GRCh38) using BWA (version 0.7.10)^25^ and the multi-sample VCF files were generated using GATK pipeline (unpublished data, available upon request). We extracted all *ACE2* variants that are located at the 15494520–15602158 positions on the X chromosome for further analysis. The Variant Effect Predictor (VEP, release 99) tool was used to annotate the selected variants through Ensembl^26^/GENCODE and RefSeq^27^ transcripts databases.

### Variation effect on the ACE2 and TMPRSS2 genes expression

The impact of expression quantitative trait loci (eQTL) variants on the expression of *ACE2* and *TMPRSS2* genes in various tissues was surveyed via the Genotype-Tissue Expression (GTEx) portal^28^.

### Three-dimensional (3D) structure and sequence of the macromolecules

Here, we focused on the interaction of viral Spike protein, TMPRSS2, B(0)AT1 with the human ACE2 protein. All of the sequence alignments were done by the NCBI/Blast server^29^; the key sites in the receptor structure were searched in the UniProt database^30,31^; all files in PDB (Protein Data Bank) format were obtained from the RCSB PDB database^32^; the protein sequences obtained from NCBI database^33^. All figures of structures and complexes have been created with the UCSF Chimera software^34^.

### Homology modeling and molecular dynamics

Although the experimentally resolved structures of S protein are available, missing domains, residues, and disulfide bonds are present in these structures. For example, RBD residues 444–448, 455–490, and 501–502 are missing in PDB: 6VSB. The primary geometry of the Spike protein monomer has been achieved by the homology modeling. We used chain A of the PDB file of 6VSB (Spike ectodomain structure), chain E of the 6M17 (Spike binding domain structure), and Spike protein (YP_009724390.1) sequence as the input structures and sequence for Modeller software^8,35^; moreover, we did loop refinement step. Because of numerous loops in the obtained structures, we observed some unacceptable conformation in the outputs where the loops tide together. Finally, we found a suitable input 3D structure for docking simulation after implementing the OPLSAA force filed, 10,000 STEP EM (emtol = 0.001 and emstep = 0.01), 500,000 NVT steps and 500,000 NPT steps both with dt = 1 fs, and, finally, 1 ns MD with dt = 2 fs on the loop-refined output of Modeller by Gromacs-2019 software. Here, homology modeling was applied by the SWISS-MODEL server^36^ to find the 3D structure of the trimeric viral Spike protein. For the TMPRSS2 protease, homology modeling was applied by the SWISS-MODEL server. To this end, the sequence of TMPRSS2 (NP_005647) was used as the primary sequence (The selected templates by the SWISS-MODEL server was 5CE1 PDB file)^37^. Moreover, the structure of B(0)AT1 protein bound to the ACE2-Spike complex was extracted from the PDB file of 6M17^38^. It worth mentioning that recently an all-atom fully-glycosylated, full-length Spike protein structure model has been released by Woo et al.^39^; their model confirms that there is not any glycation site on the interface of the Spike protein towards the ACE2.

### Binding sites and key residues of macromolecules

Although there is not enough information about the active site and the catalytic site of TMPRSS2, it is a trypsin-like protease. Thus, we compared the TMPRSS2 sequence (NP_005647) and its structure with the trypsin 2AGE PDB file and the corresponding FASTA sequence. The 3D structure of TMPRSS2 obtained from the SWISS-MODEL was superimposed to the 3D structure of the trypsin (2AGE PDB file). Catalytic triad and active sites of trypsin, as well as the cleavage site of the ACE2, were acquired based on the previous studies. His57, Asp102, and Ser195 constitute the catalytic triad of trypsin and residues Asp189, Ser190, and Gly219 are responsible for the substrate interactions and positioning^40–42^. Additionally, it was reported that the arginine and lysine residues within the region 697–716 as well as 652–659 of ACE2 are required for the cleavage of the ACE2 receptor by TMPRSS2^15^. Besides, to perform docking simulation for characterizing the binding sites of ACE2 to the viral Spike protein, we used the previous studies^35,43,44^ and also analyzed the PDB file of 6M17 (SARS-CoV-2 Spike/ACE2-B0AT1 complex) by the LigPlot^+^ tool^45^.

### Docking and analyzing the results

Thanks to the High Ambiguity Driven protein–protein Docking (Haddock) server available at https://haddock.science.uu.nl/^46,47^, the possible interactions between all the proteins of interest were simulated. The results were analyzed employing LigPlot^+^ and UCSF-Chimera. To obtain the optimal docking parameters, the interactions of the wild-type receptor with the partners were simulated using different parameters.

### Evaluation of the *ACE2* variants on the protein–protein interaction

To determine the impact of *ACE2* variants on the molecular mechanism of the virus pathogenesis process in Iran, all missense variants found within our population were considered. The residue corresponding to each missense variant and its position in the 3D structure was determined. According to the obtained positions, the variants that possibly affect the viral Spike protein binding to the ACE2 receptor were specified and each mutation was separately applied to the ACE2 3D structure using Modeller 9.23 software^48^. Then the interaction of the mutated protein with its partner was simulated by docking simulation.

### Ethics approval

All procedures were under the ethical standards of the ethics committee on human subject research at Research Institute for Endocrine Sciences, Shahid Beheshti University of Medical Sciences (code of “IR.SBMU.ENDOCRINE.REC.1395.366”) and with the 1964 Helsinki Declaration and its later amendments or comparable ethical standards.

### Consent to participate

Informed written consent had been obtained from all participants. The study was approved by the ethics committee of the Research Institute for Endocrine Sciences.

### Consent for publication

As corresponding author, I confirm that the manuscript has been read and approved for submission by all the named authors. We declare that this manuscript is original, has not been published before, and is not currently being considered for publication elsewhere.

## Results

### *ACE2* genetic polymorphisms within the Iranian population

**Figure 1:**
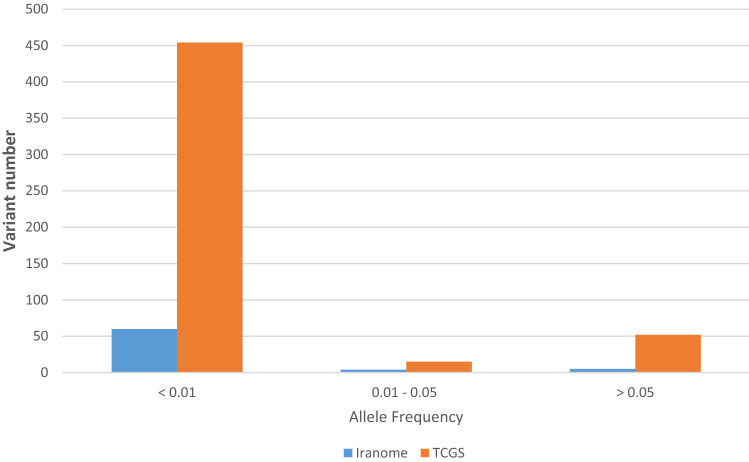
Allele frequency spectrum of *ACE2* variations within the Iranian population (TCGS and Iranome datasets).

**Figure 2:**
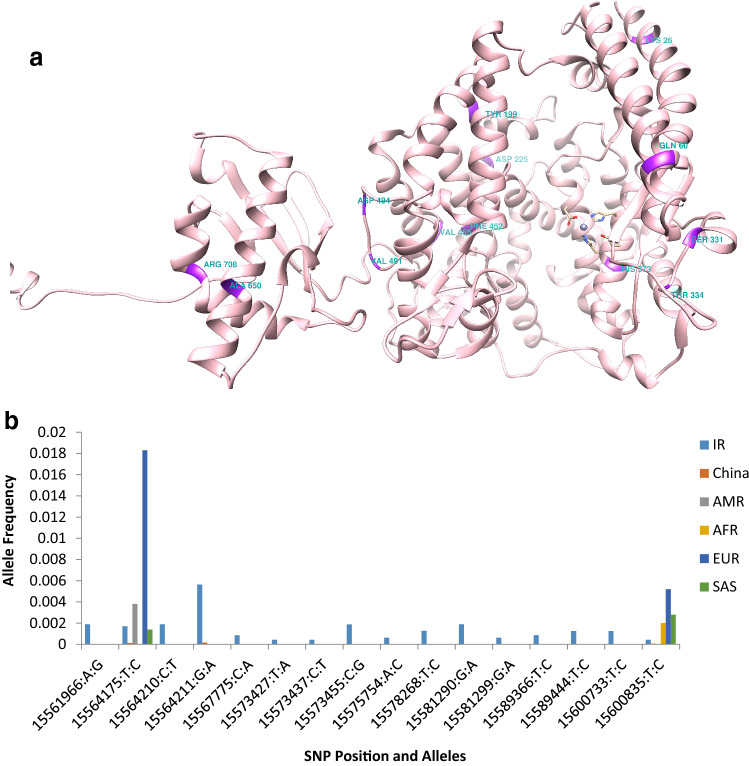
(**a**) The position of missense variants on the 3D structure of ACE2 receptor. The residues of 26 and 331 located at the ACE2 binding site to viral Spike protein, the residue 373 is in the vicinity of the zinc ion binding residues, and residues of 708 and 650 are in the binding site of the ACE2 to TMPRSS2. This figure has been produced by the UCSF Chimera 1.14-linux_x86_64 (https://www.cgl.ucsf.edu/chimera/download.html). (**b**) Missense *ACE2* variants in Iran, China, and 1000 Genome Project populations.

In the present study, a total of 570 genetic variations, including single nucleotide polymorphisms (SNP) and insertion/deletion (INDEL) were detected in the Iranian population. The allele frequency (AF) analysis of the variants demonstrated that most of them have low frequency in the Iranian population and just 54 common polymorphisms detected in this population (Fig. 1). The survey of these variants in other populations showed just 192 variants with different allele frequencies shared among Iranian, China, and five super populations of the 1000 Genome Project (Supplementary File 1). The genetic polymorphisms are mostly located in the intronic region of the *ACE2* gene. In contrast, 16 genetic variants affecting the amino acid sequence of ACE2 were found within the Iranian population (Supplementary File 2). The position of missense variants on the 3D structure of the ACE2 receptor were illustrated in Fig. 2a and demonstrated their allele frequency within an Iranian population and the 1000 Genome Project population (Fig. 2b). The key genetic variations detected in the Iranian population include rs4646116 (K26R) at the binding site of ACE2 to the viral spike protein and rs769062069 (R708Q), rs776995986 (R708W), and A650S (not found in dbSNP) at the binding site of the ACE2 to TMPRSS2 protease (Supplementary File 2).

### 3D structure of the viral Spike protein, ACE2, TMPRSS2

**Figure 3:**
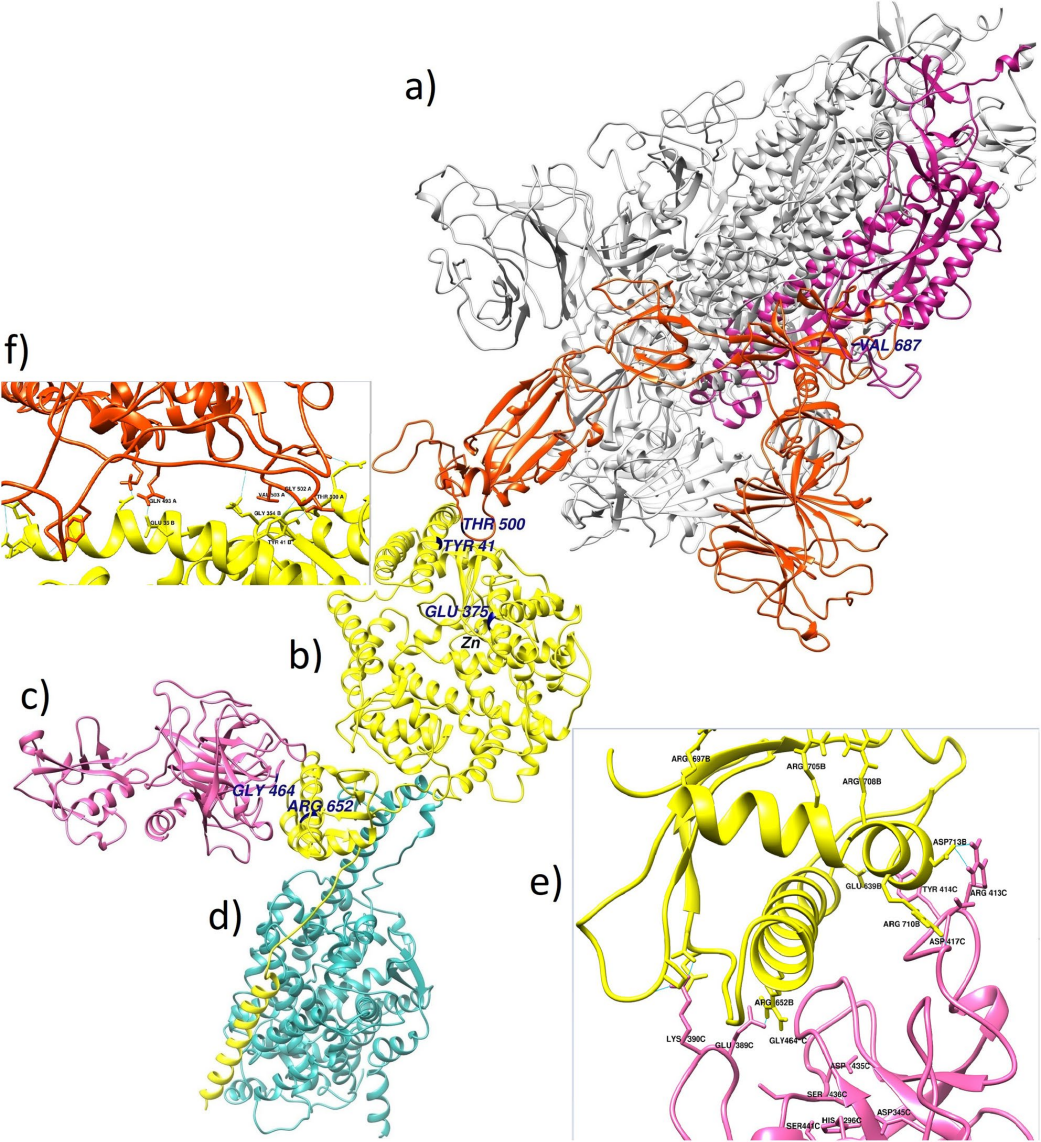
Structural alignment of the Spike trimer, Spike monomer-ACE2, ACE2-TMPS2, and ACE2-B0AT1 complexes. The whole picture of the spatial positions of the main partners of ACE2 in the pathogenic process of SARS-CoV-2. This figure has been produced by the UCSF Chimera 1.14-linux_x86_64 (https://www.cgl.ucsf.edu/chimera/download.html). (**a**) The 3D structure of the Spike protein trimer, the S1 and S2 subunits presented in orange-red and violet-red colors, respectively. (**b**) The 3D structure of the ACE2 human receptor (yellow). (**c**) The 3D structure of the TMPRSS2 protease (hot pink); (**d**) The 3D structure of the *B(0)AT1 (SLC6A19*) Amino acid transporter (light sea green); (**e**) The interface of ACE2 and TMPRSS2 proteins; Letters B and C indicate ACE2 and TMPRSS2 chain IDs, respectively. Arg652 of ACE2 has been placed in the best position relative to the triad of TMPRSS2 (His296, Asp345, and Ser441) and its key residues (Asp435, Ser436, and Gly464). (**f**) The interface of ACE2 and Spike proteins; the main residues of ACE2 and Spike involved in the interaction were shown.

The 3D structures of Spike trimer and TMPRSS2 protein obtained from the homology modeling together with the Spike monomer-ACE2 complex, and ACE2-TMPRSS2 complex resulted from the Haddock server have been presented in Fig. 3. TMPRSS2 model had a sequence identity of 33.82, the sequence similarity of 0.38, sequence coverage of 0.70, and a QMEAN of -2.65^49^. QMEAN (Qualitative Model Energy Analysis) is “QMEAN Z-score” which provides an estimate of the "degree of nativeness" of the structural features observed in the model on a global scale and scores of -4.0 or below are an indication of models with low quality; thus the QMEANS of -2.65 confirms the validity of our models. The structural alignment of the Spike trimer, Spike monomer-ACE2, ACE2-TMPRSS2, and ACE2-B(0)AT1 complexes created an overall view of the ACE2 receptor and its main partners as shown in Fig. 3.

### Homology modeling and finding the key residues of TMPRSS2

**Figure 4:**
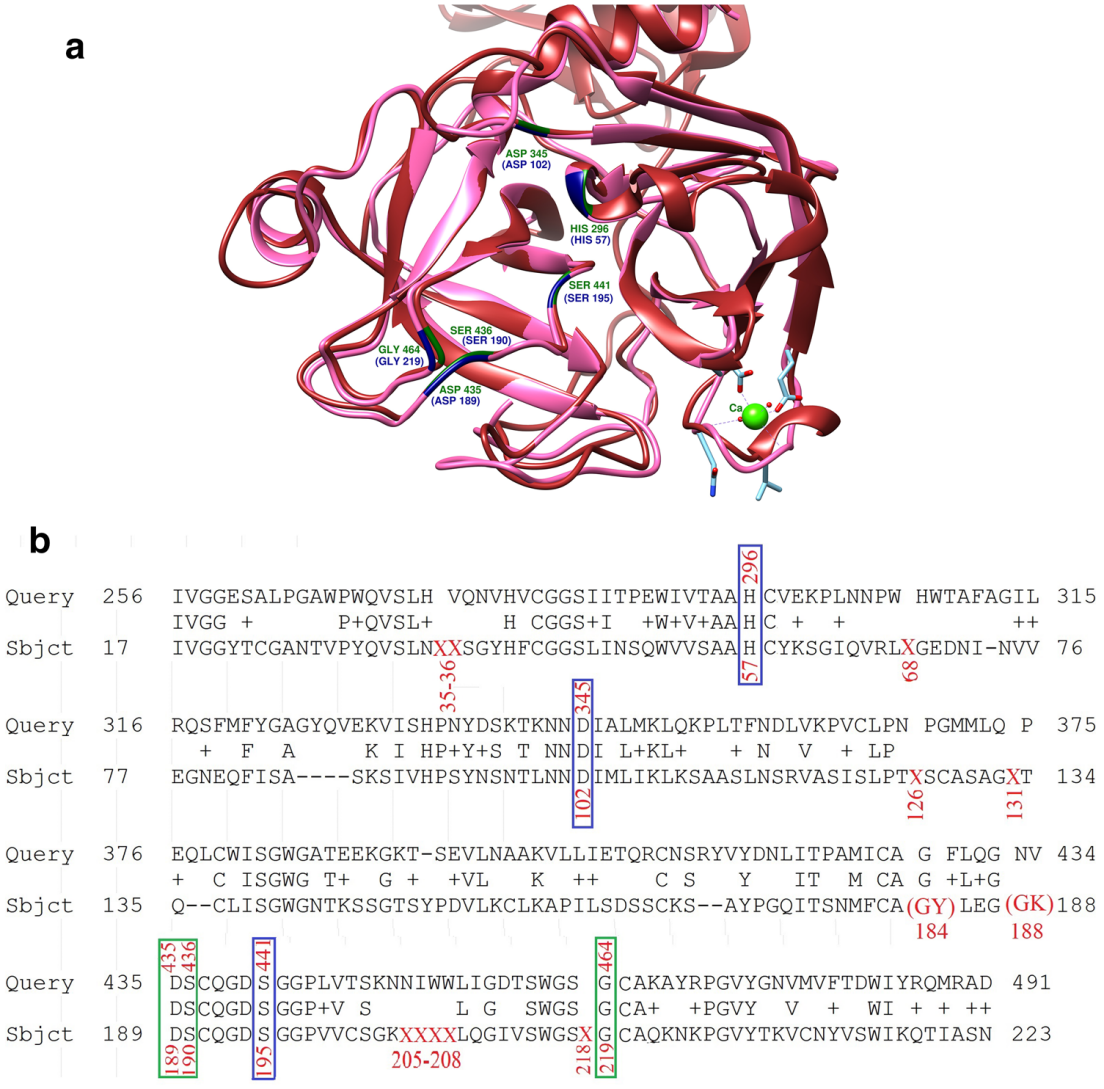
3D structure and sequence alignment of TMPRSS2 against the Trypsin. (**a**) The 3D structure of TMPRSS2 is colored in brown and the key residue in dark-green, the 3D structure of Trypsin is in hot pink color and its key residue in navy-blue. This figure has been produced by the UCSF Chimera 1.14-linux_x86_64 (https://www.cgl.ucsf.edu/chimera/download.html). (**b**) The pairwise alignment of NP_005647(TMPRSS2) with the 2AGE sequence of trypsin has been shown. The important matched residues have been put in rectangular (the catalytic triad in blue and important residue for binding in green). The missing residues in the PDB file have been shown by X and the double residues in one position put in parentheses.

The 3D structures of viral Spike protein and TMPRSS2 obtained by homology modeling are shown in Fig. 3a,c. With further investigation of the TMPRSS2 sequence alignment and the structural superimposition of obtaining 3D structure against the trypsin, we recognized that there are some missing positions in both the trypsin PDB file (2AGE) and the corresponding FASTA sequence as they were compared to the reference sequence. In detail, the positions of 35, 36, 68, 126, 131, 205–208, and 218 are missing within the structural and sequence files. 2AGE PDB comes from the crystallography of bovine (*Bos taurus*) trypsin so, we checked these missing positions at the *Bos taurus* reference sequence (NP_001107199.1); unexpectedly, they were not present within this sequence. Therefore, we aligned NP_001107199.1 against the human trypsin reference sequence (NP_001184027.1); again, no insertion or deletion was observed at the aforementioned positions in the human sequence. Moreover, two residues were found in the positions of 184 and 188 (Gly, Tyr, and Gly, Lys) of PDB file while they present as two different residues at two independent positions of the bovine reference sequence (Fig. 4b). Considering the changes in the residue numbers due to the missing positions as well as the positions of 184 and 188, the residues of His296, Asp345, Ser441, Asp435, Ser436 and Gly464 at TMPRSS2 were matched to the residues of His56, Asp100, Ser193, Asp187, Ser188, and Gly212 of human trypsin, respectively. However, without considering the above-mentioned issues, in line with the previous studies^41,42^, these residues are matched to the residues of His57, Asp102, Ser195, Asp189, Ser190 and Gly219 of the trypsin PDB file, respectively (Fig. 4). Taken together, the catalytic triad of TMPRSS2 protease is composed of His296, Asp345, and Ser441 and its key residues in the binding site of the protease are Asp435, Ser436, and Gly464.

### Docking results and the 3D structure of complexes formed with ACE2

**Figure 5:**
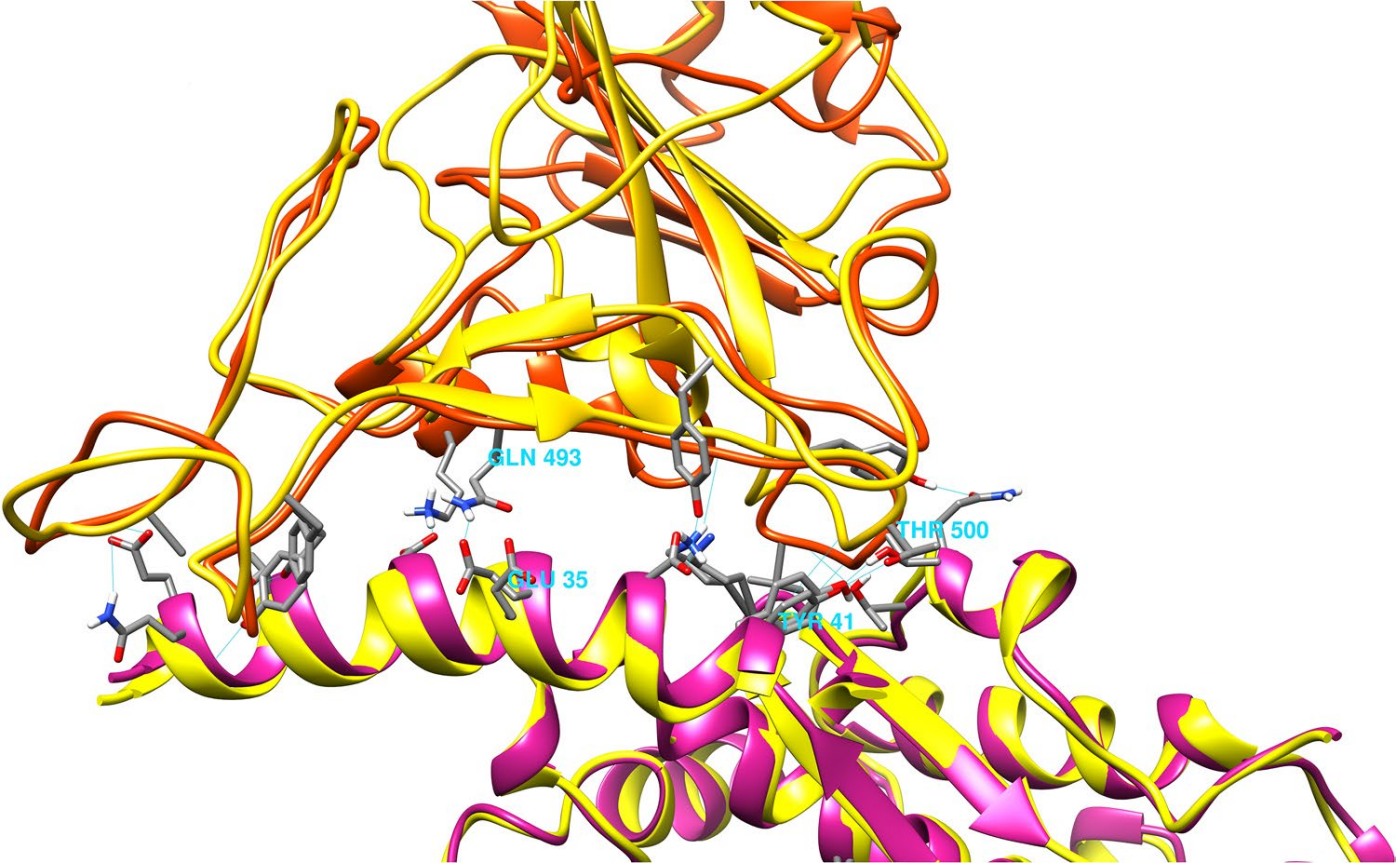
The superimposition of the experimental PDB and our result for Spike-ACE2 interaction. The superimposed experimental 3D structure of the ACE2-Spike complex (6M17 PDB file) with the simulated complex have been shown in red and yellow, respectively. This figure has been produced by the UCSF Chimera 1.14-linux_x86_64 (https://www.cgl.ucsf.edu/chimera/download.html).

The primary geometry of the Spike protein monomer has been achieved by the homology modeling. Chain A of the PDB file of 6VSB (Spike ectodomain structure) and chain E of the 6M17 (Spike binding domain structure) were used as the input structures for Modeller software and also performed the loop refinements. The energy minimization and molecular dynamics were done by Gromacs-2019 software^50,51^. The complexes resulted from the interaction of Spike monomer and TMPRSS2 proteins with the wild-type ACE2 have been shown in Fig. 3e,f. Furthermore, the superimposing of the experimental and simulated Spike-ACE2 complexes has been shown in Fig. 5.

### TMPRSS2 interaction sites and its cleavage site on ACE2

To scrutinize the previous results^15^ about the cleavage site of the ACE2 targeted by the TMPRSS2, we run three different docking analyses by three different input active residues using the Haddock server. In the Haddock algorithm, the active residues are restrained to be part of the interface throughout the simulation, if possible, otherwise incurring in a scoring penalty^47^. We considered the Arg708, Arg710, and Arg652 as active residues, separately, and run three different ACE2-TMPRSS2 dockings. Our results were also surveyed by the LigPlot^+^ software as illustrated in Supplementary Fig. S1a–c. The hydrogen bond between the Arg652 of ACE2 and the Glu389 of TMPRSS2 appears in the all dockings results. As the 3D conformation of the interface of ACE2-TMPRSS2 (Fig. 3e) demonstrated, the Glu389 of TMPRSS2 is located at the same groove as the residues Asp435 and Gly464 of this protease. Furthermore, based on the Haddock results presented in Table 1, the best score (the most negative Haddock score) and the largest cluster size was obtained when the Arg652 was selected as the active residue for docking analysis. Consequently, the Arg652 of ACE2 is in the best position to interact with the catalytic site residues of the TMPRSS2. Therefore, the obtained results support Arg652 as the cleavage site of ACE2 targeted by the TMPRSS2 protease while the previous studies had also been introduced to the Arg652 and the arginine and lysine residues within regions of 697–716 as the receptor cleavage site^15,52^. However, our results support the essential role of this region on the proper orientation of TMPRSS2-ACE2 where Arg710 of the receptor interacts with the Asp454 and Tyr453 of TMPRSS2.

**Table 1:** Docking results of the wild-type and mutated ACE2 receptor with TMPRSS2.

	Haddock score	Clustered structures	Cluster size	z-score	Hydrogen bonding (n)
TMPRSS2-ACE2 (wild type; active residue = ARG 710)	− 93.9 ± 2.5	254	104	− 1.9	10
TMPRSS2-ACE2 (wild type; active residue = ARG 708)	− 92.2 ± 5.9	246	128	− 1.8	11
TMPRSS2-ACE2 (wild type; active residue = ARG 652)	− 106 ± 6	275	195	− 1.9	9
TMPRSS2-ACE2(ALA650>SER; Active residue = ARG 652)	− 98.3 ± 10.8	253	108	− 1.7	10
TMPRSS2-ACE2(ARG708>GLN; active residue = ARG 652)	− 92 ± 9.2	259	96	− 1.8	12
TMPRSS2-ACE2(ARG708>TRP; active residue = ARG 652)	− 94.4 ± 6.2	253	137	− 2.5	12

The column of “Haddock score” in this table shows the score of the best-obtained complex, the column “Clustered structures” is the number of complexes that finally clustered by the haddock server (we set the initial docking number = 10,000 and the number of structure for final analyses = 300) and Cluster size represents the population size of the cluster of the best-obtained complex and the final column is the z-score of this complex among this cluster.

Indeed, the various studies investigated the possibility of forming the B(0)AT1-ACE2-TMPRSS2 complex as it is not clear whether the TMPRSS2 can bind to ACE2-B(0)AT1 complex. To address this issue, all interactions have been presented in details in Supplementary Fig. S2. By comparing Fig. S1c and Fig. S2a,b, it is found that the ACE2 and TMPRSS2 interaction was not dependent on the B(0)AT1/ACE2 interaction. To explicitly survey the result of the interaction between TMPRSS2 and the ACE2-B(0)AT1 complex, the conformation of B(0)AT1-ACE2-TMPRSS2 complex was obtained from docking simulation of the ACE2-B(0)AT1 and TMPRSS2, whereas the ACE2-B(0)AT1 has been extracted from the experimental 3D structure (6M17 PDB file). According to our results, there is not any spatial restriction or conflict between ACE2 partners (B(0)AT1 and TMPRSS2). Therefore, TMPRSS2 can bind to ACE2-B(0)AT1 complex as it can interplay with ACE2 in the absence of B(0)AT1 (Fig. S3a,b). Likewise, based on our results presented in Fig.S2b, the residues involved in ACE2 dimerization can conflict with the ACE2-TMPRSS2 interaction. Especially, Arg710, Ala 714, and Gln653 of each ACE2 monomers bind to the other monomer. Therefore, ACE2 dimerization and the consequent spatial constraint can impede the access of TMPRSS2 to the ACE2 receptor.

### Missense variants impact on binding of ACE2 receptor to the viral Spike protein

**Figure 6:**
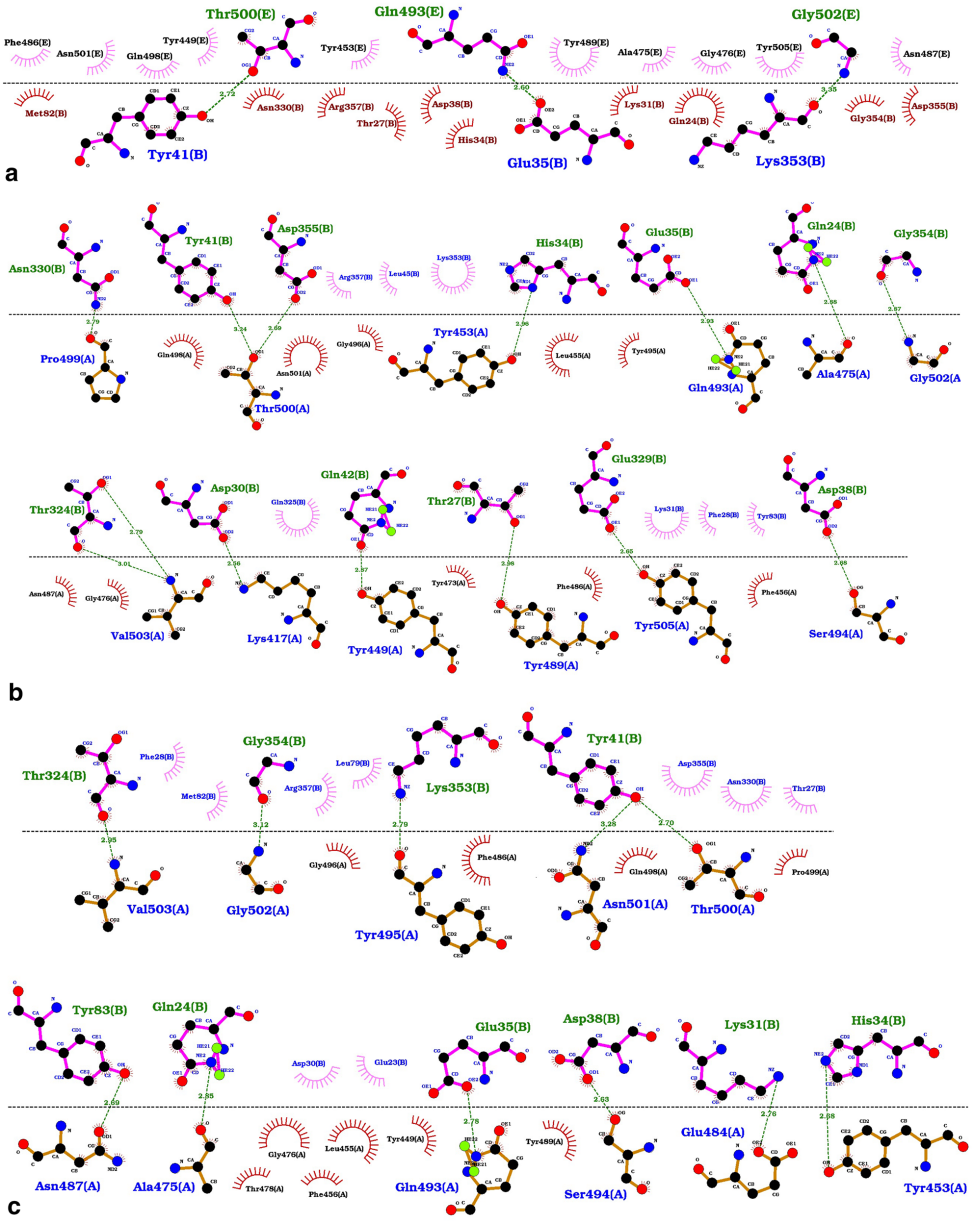

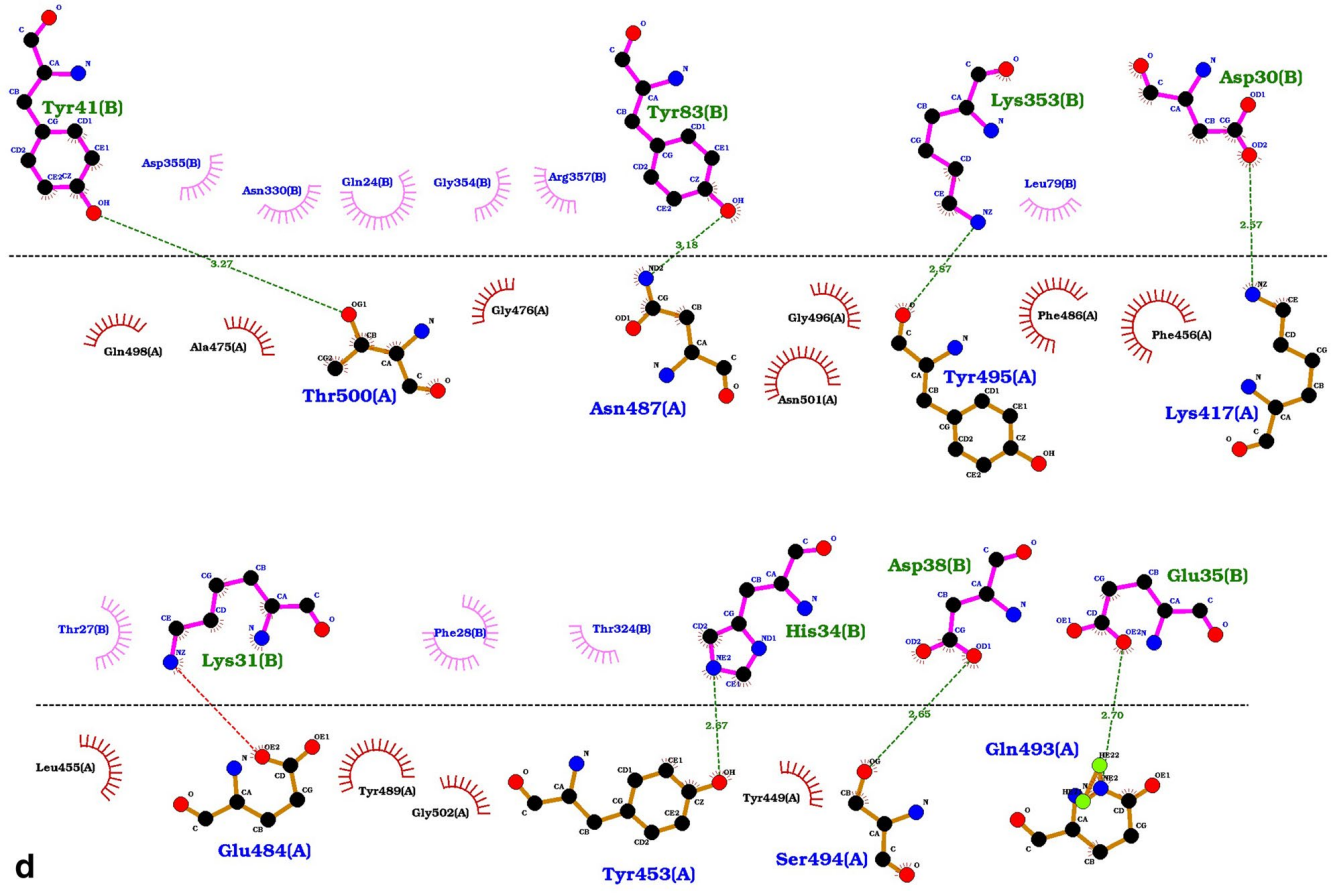
Spike-ACE2 interaction details. The hydrogen and hydrophobic bonds within different ACE2-Spike protein complexes. The ACE2 residues have been labeled by “B” letter and the Spike protein residues have been shown by “A”. The green line represents the hydrogen bonds. Residues involved in hydrogen bonds have been shown in blue and green colors, while brown and black colors are used for hydrophobic interactions. The length of the hydrogen bonds is determined by the angstrom. (**a**) Experimental 6M17chainB-6M17chainA complex; (**b**) Simulated Spike-ACE2 complex; (**c**) Simulated Spike-ACE2(Lyz26 >Arg) complex; (**d**) Simulated Spike-ACE2(Ser331 >Phe) complex.

Firstly, the interaction of wild-type ACE2 structure (chain B of 6M17 PDB file) with the 3D structure of Spike monomer protein was simulated using the Haddock server. Figure 5 shows the superimposition of the experimental structure and our result (Fig. 5). Additionally, both hydrogen and hydrophobic bonds within different ACE2-Spike protein complexes were recognized using the LigPlot^+^ software as presented in Fig. 6. Interestingly, two hydrogen bonds, Thr500 (Spike)/Tyr41 (ACE2) as well as Gln493 (Spike)/Glu35 (ACE2) were common between the experimental (Fig. 6a) and our simulated Spike-ACE2 complex. But, the interaction between Gly502 of Spike protein and Lys353 of ACE2 was not observed; instead, we detected the hydrogen bond between the Gly502 of Spike protein and the Gly354 of ACE2 receptor (Fig. 6b).

Among the missense variants found in the Iranian population, the K26R and S331F are located at the binding site of the ACE2 receptor of the viral Spike protein. The corresponding mutations were separately applied and the interaction of the mutated receptor structure with the 3D structure of the Spike monomer was simulated using the Haddock server and the results shown in Table 2. According to the further analysis by the LigPlot^+^ software, it was interestingly found that the two aforementioned common hydrogen bonds were also present in both the mutated ACE2-Spike complex (Fig. 6c,d). Furthermore, besides the two common hydrogen bonds, the interaction between the His34 of ACE2 with Tyr453 of Spike along with Asp38 of ACE2 with the Ser494 Spike was also observed in all wild-type and mutated ACE2/Spike complexes.

**Table 2:** Docking analysis of the wild-type and mutated ACE2 structure with the Spike monomer protein.

	Haddock score	Clustered structures	Cluster size	z-score	Number of Hydrogen bonding
Spike-ACE2 (wild type)	− 136.0 ± 6.1	167	115	− 2.2	14
Spike-ACE2(LYZ26>ARG)	− 127.8 ± 2.8	153	95	− 1.8	11
Spike -ACE2(SER331>PHE)	− 122.5 ± 1.8	165	96	− 1.9	8

The column of “Haddock score” in this table shows the score of the best-obtained complex, the column “Clustered structures” is the number of complexes that finally clustered by the haddock server (we set the initial docking number = 2000 and the number of structure for final analyses = 200) and Cluster size represents the population size of the cluster of the best-obtained complex and the final column is the z-score of this complex among this cluster.

According to Fig. 6c,d, four hydrogen bonds always present in the wild-type and mutated ACE2 receptor, hence, mutations at the corresponding positions cannot adversely affect the receptor binding to the viral Spike protein. But, the K26R and particularly the S331F mutations can decrease the Haddock score, cluster size, as well as the number of hydrogen bonds between the ACE2 receptor and the Spike protein (Table 2). Consequently, both mutations can slightly reduce the affinity of the receptor for the viral Spike protein.

### Missense variants impact on the ACE2 cleavage

We observed three missense variants, Ala650Ser, rs769062069 (Arg708Gln), and rs776995986 (Arg708Trp) at the ACE2 receptor. As shown in Fig. 2b, rs776995986 was a shared variant only between Iran and China population, while rs769062069 and A650S were found to be specific in the Iranian population; the allele frequency of all variations was lower than 0.01 (Fig. 2b). The key role of these positions for ACE2 receptor interaction with TMPRSS2 protease as well as their low-frequencies in our population motivated us to examine how these polymorphisms can influence the ACE2 cleavage. To this end, the Arg708Gln, Arg708Trp, and Ala650Ser mutations were separately applied to the ACE2 structure and the interaction of the mutated receptors with the 3D structure of TMPRSS2 was simulated and compared with the wild-type receptor (Table 1). We analyzed the ACE2-TMPRSS2 interaction in detail by the LigPlot^+^ tool as the results presented in Fig. S1d–f. As the Fig. S1 demonstrates, the three hydrogen bonds, Arg652(ACE2)/GLu426(TMPRSS2), Arg710(ACE2)/Tyr453(TMPRSS2), and Arg710(ACE2)/Asp454(TMPRSS2) were always observed in the wild-type and mutated TMPRSS2 structures. However, the weaker Haddock score and the smaller cluster size have been resulted from all three applied mutations on TMPRSS2, suggesting the reduced affinity of TMPRSS2 for the cleavage of ACE2.

### Impact of genetic variants on *ACE2* and *TMPRSS2* genes expression

To investigate the impact of genetic variants on *ACE2* and *TMPRSS2* gene expression in various tissues, the GTEx portal was used. As previously described^24^, the *ACE2* expression in 20 tissues can be regulated by 15 unique eQTL variants with q-value < 0.05. Further investigation showed that all of these variants except for rs112171234, rs12010448, rs75979613, rs143695310 are shared variants between Iran, China, and the 1000 Genome Project populations with allele frequency > 0.05, which were substantially higher in China and EAS populations (Supplementary File 3). However, it was noted that all of these eQTL variants affected the *ACE2* expression in the nervous tissues, mostly brain, not in the SARS-CoV infection-related main tissues, including lung, kidney, and intestine. Similarly, it found that 203 eQTL variants with the q-value < 0.05 influencing the *TMPRSS2* expression in five tissues. The lung, testis, and prostate tissues allocated the highest number of variants, respectively. According to the normalized effect size (NES) provided at the GTEx portal, 76 variants can increase the *TMPRSS2* expression in the lung tissue (Supplementary File 4). we surveyed the allele frequency of these variants in all populations of the 1000 Genome project as well as the Iranian population, which can be found in Supplementary File 4. Interestingly, almost all variants had the highest allele frequency in Iranian and European populations while the lowest allele frequency variants were recognized in East Asian populations. Therefore, homozygous genotype forms of these variants appear to enhance the host susceptibility to SARS-CoV-2 infectivity and pathogenesis via enhancing the *TMPRSS2* expression.

## Discussion

In this study, the SARS-CoV-2 Spike protein/ACE2 receptor interaction as well as the receptor cleavage by the TMPRSS2, which is necessary for the virus pathogenesis were simulated. Furthermore, the various *ACE2* polymorphisms and the impact of key *ACE2* variants within the Iranian population on the susceptibility to COVID-19 were investigated through homology modeling and simulation methods. We acquired the RBD-Spike/ACE2 complex, which was high consistent with the experimentally obtained 3D structure. The recently determined full-length human ACE2 revealed that the receptor can be in complex with B(0)AT1, and the ACE2-B(0)AT1 structure can simultaneously bind to two Spike viral proteins^38^. It has also been speculated that the TMPRSS2 access to the cleavage site of ACE2 may be hampered by the B(0)AT1. However, we found that the ACE2 dimerization may interfere with the TMPRSS2 function as the cutting site of ACE2, specific residues 697–716 and 652 located at the dimeric interface of ACE2 and are not available for the protease. Additionally, the *ACE2* transcripts are detected in several tissues, especially those related to the COVID-19, including lung, heart, and adipose tissues where B(0)AT1 is not expressed, proposing the wider *ACE2* expression pattern as compared to B(0)AT1^3^. Therefore, B(0)AT1 might not be able to prevent the ACE2 cleavage and the subsequent SARS-CoV-2 infection at least in the lung, heart, and adipose tissues.

It hypothesized the diverse allele frequency of missense variants found in the Iranian population, especially the variants located at or near the ACE2 binding site can influence the varied populations' susceptibility to the SARS-CoV-2 infection. To test this hypothesis, we separately simulated the Spike protein interaction with the two mutant ACE2 receptors at the K26R and S331F residues. The interaction of wild-type and mutant receptors with Spike protein was quantified through calculating the Haddock score and the hydrogen bonds number. Our findings implied that both mutations, specifically S331F can slightly decrease the affinity of the ACE2 receptor to the SARS-CoV-2 Spike protein. In agreement with our results, Calcagnile et al. also reported the similar effect of the K26R variant on the viral Spike protein binding to the ACE2 receptor^3^. While K26R was found in other populations, including African, European, and South Asian populations of the 1000 Genome Project, the S331F polymorphism appears to be specific for the Iranian population as we could detect it neither in the 1000 genome populations nor in the genome aggregation database (gnomAD). Therefore, in addition to the variety of factors, like sex and ACE2 inhibitor drugs, the *ACE2* polymorphisms can modify the infection susceptibility through the alternation of receptor affinity to the viral Spike protein^53^. It has been recently revealed that the SARS-CoV-2 cell entry depends on both ACE2 and TMPRSS2 functions and can be impaired using the protease inhibitors^8^. The cleavage of the ACE2 C-terminal segment by TMPRSS2 protease facilitates viral entry into the host cell and plays a critical role in the virus pathogenesis process.

In agreement with the previous studies^41^, we recognized His296, Asp345, and Ser441 as the catalytic triad of TMPRSS2 as well as Asp435, Ser436, and Gly464 as the key residues at the protease binding site. Afar et al. described that Ser441Ala mutation leads to the loss of TMPRSS2 activity, which further support our results^54^. Based on the study conducted by Heurich et al., the arginine and lysine residues within ACE2 region 697 to 716 are necessary for its cleavage by TMPRSS2. Moreover, they revealed that ACE2 shedding by another protease, ADAM17, depends on the arginine and lysine residues within the ACE2 region 652–659^15^. Our results also confirmed the main role of ACE2 region 697–716, especially, the Arg710 for establishing the ACE2-TMPRSS2 complex. Furthermore, it is found that this interaction facilitates the proper binding of the ACE2 receptor to the TMPRSS2 protease and Arg652 as the main cleavage site of ACE2 can be targeted by the TMPRSS2, in contrast to Heurich et al. research, which considered the Arg652 residue as the ADAM17 cleavage site.

As docking results (Fig. 3e) displayed, the Arg695, Arg705, and Arg708 are not accessible by the TMPRSS2 protease while Arg652 is located at the appropriate position toward the protease catalectic triad. It is also observed that Arg716 was presented at the binding site of ACE2 and Arg710 always contributes to creating the hydrogen bonds, proposing its important role in mediating the proper protease-receptor interaction (Fig. 6).

It has been recently reported the co-expression of *ACE2* and *TMPRSS2* in the respiratory and digestive tracts is critical for SARS-CoV-2 entry to the host cell^52,55^. Furthermore, the enhanced *ACE2* transcript level in lung tissue was observed with increasing age, especially in men, which might be resulted in increased infection susceptibility or the greater severity of disease^56^. Considering the *ACE2* location on the X chromosome and male hemizygosity for this gene, the presence of risk allelic variants inducing *ACE2* expression can lead the higher ACE2 expression in all cells, which may further explain the more disease susceptibility in men^2^. However, by investigating the GTEx portal, any risk eQTL variant affecting the *ACE2* expression in lung, kidney, and intestine tissues could not be recognized. In contrast, it was found that the *TMPRSS2* expression was positively modulated by multiple QTL variants in lung tissue. The highest allele frequency of these variants within Iranian and European populations as well as the lowest allele frequency in individuals with East Asian ancestry can somewhat account for the discrepant disease predisposition and mortality rate across populations. Consequently, the genetically different response or vulnerability to the SARS-CoV-2 infection is expected across populations or even individuals.

## Conclusion

In summary, surveying the effect of critical *ACE2* mutations found within the Iranian population on its interaction with the viral Spike protein and TMPRRS2 demonstrated two mutations, K26R and S331F, which the latter was currently only detected in the Iranian population, are capable to reduce the receptor affinity for the viral Spike protein. Moreover, there are multiple eQTL variants with the highest allele frequency in Iranian and European populations and the lowest allele frequency in the East Asian population, which positively regulated the *TMPRSS2* expression in lung tissue. Taken together, genetic variations have the potential to reshape the viral pathogenicity and disease susceptibility across populations; thus, we need to consider the genetic variations on *ACE2* as well as other genes for designing the efficient drugs and vaccines.

## Supplementary Information

Supplementary Information 1.

Supplementary Information 2.

## Data Availability

The data available as supplementary files and if additional information is required, it is possible to send more details.
