# Supplementary material for: SARS-CoV-2 infection susceptibility influenced by ACE2 genetic polymorphisms: insights from Tehran Cardio-Metabolic Genetic Study
Source: Sci Rep. 2021 Jan 15;11:1529. doi: 10.1038/s41598-020-80325-x (PMC7810897; doi:10.1038/s41598-020-80325-x)
Supplement: Supplementary file 1 — Supplementary Information 1. [file 41598_2020_80325_MOESM1_ESM.docx]

**SARS-CoV-2 infection susceptibility influenced by ACE2 genetic polymorphisms: insights from the** **Tehran Cardio-Metabolic Genetic Study**

Hossein Lanjanian,^1,9^ Maryam Moazzam-Jazi,^2,9^ Mehdi Hedayati,^3^ Mahdi Akbarzadeh,^4^ Kamran Guity,^5^ Bahareh Sedaghati-khayat,^6^ Fereidoun Azizi,^7^ Maryam S Daneshpour,^8*^

**Authors**

1. Hossein Lanjanian, Ph.D., Cellular and Molecular Endocrine Research Center, Research Institute for Endocrine Sciences, Shahid Beheshti University of Medical Sciences, Tehran, Iran. Email: H.Lanjanian@ut.ac.ir; ORCID: **0000-0003-4284-6592**
2. Maryam Moazzam-Jazi, Ph.D., Cellular and Molecular Endocrine Research Center, Research Institute for Endocrine Sciences, Shahid Beheshti University of Medical Sciences, Tehran, Iran. Email: setareh227@gmail.com; ORCID: **0000-0003-3603-9284**
3. Mehdi Hedayati, Ph.D., Cellular and Molecular Endocrine Research Center, Research Institute for Endocrine Sciences, Shahid Beheshti University of Medical Sciences, Tehran, Iran. Email: [hedayati@endocrine.ac.ir](mailto:hedayati@endocrine.ac.ir); ORCID: **0000-0001-5816-775X**
4. Mahdi Akbarzadeh, Ph.D., Cellular and Molecular Endocrine Research Center, Research Institute for Endocrine Sciences, Shahid Beheshti University of Medical Sciences, Tehran, Iran. Email: akbarzadehms@sbmu.ac.ir; ORCID: **0000-0002-8048-744X**
5. Kamran Guity, MSc, Cellular and Molecular Endocrine Research Center, Research Institute for Endocrine Sciences, Shahid Beheshti University of Medical Sciences, Tehran, Iran. Email: kamran.guity@decode.is; ORCID: **0000-0002-8379-9668**
6. Bahareh Sedaghati-khayat, MSc, Cellular and Molecular Endocrine Research Center, Research Institute for Endocrine Sciences, Shahid Beheshti University of Medical Sciences, Tehran, Iran. Email: [b.sedaghatikhayat@erasmusmc.nl](mailto:b.sedaghatikhayat@erasmusmc.nl); ORCID: **0000-0002-7665-8648**
7. Fereidoun Azizi, Endocrine Research Center, Research Institute for Endocrine Sciences, Shahid Beheshti University of Medical Sciences, Tehran, Iran. Email: azizi@sbmu.ac.ir; ORCID: **0000-0002-6470-2517**
8. Maryam S Daneshpour, Ph.D., Cellular and Molecular Endocrine Research Center, Research Institute for Endocrine Sciences, Shahid Beheshti University of Medical Sciences, Tehran, Iran. Email: daneshpour@sbmu.ac.ir; ORCID: **0000-0003-1525-8672**
9. These authors contributed equally to this work

**
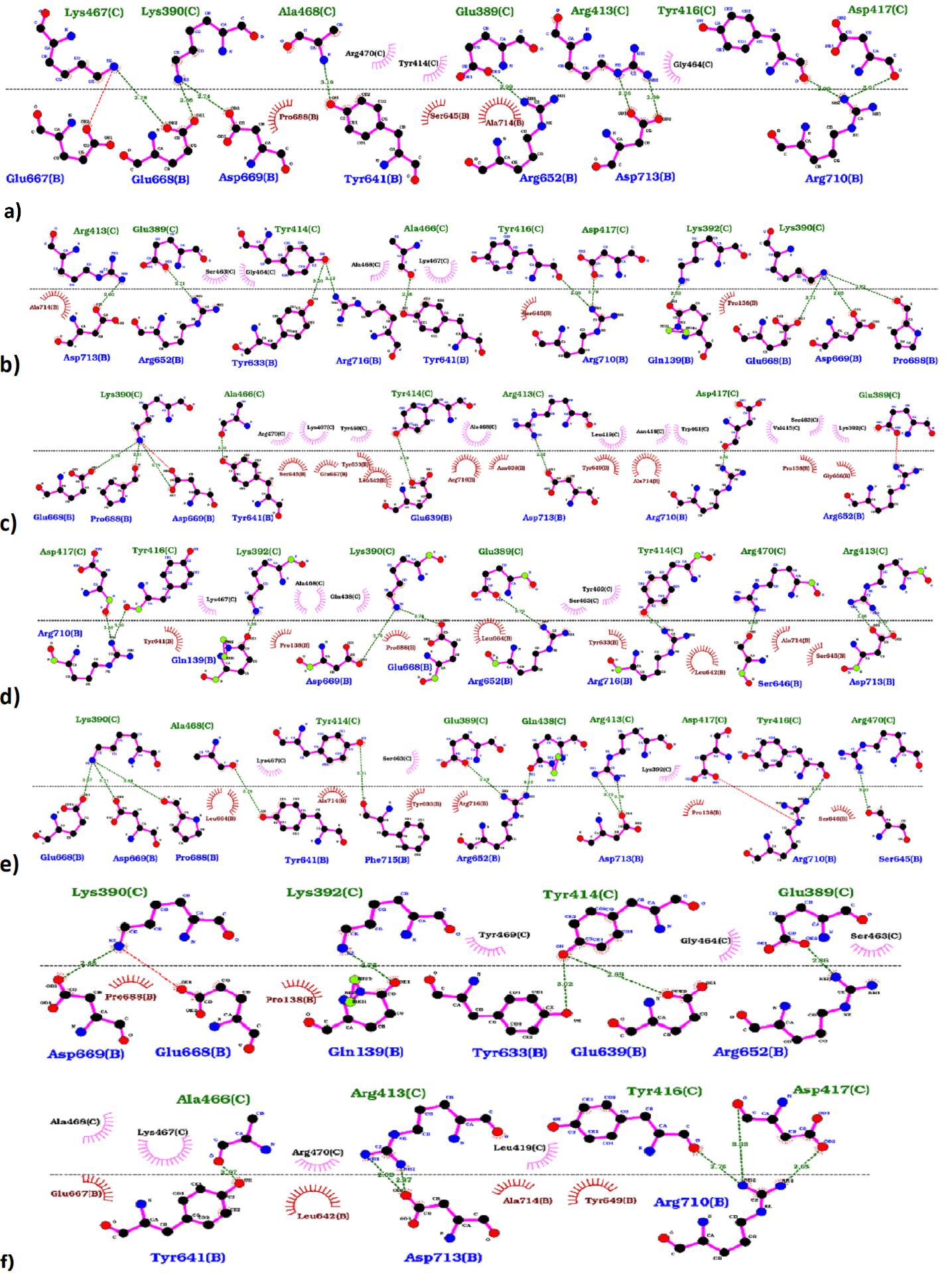
**

**Fig.S1 ACE2-TMPRSS2 interaction details**

The details of ACE2(wild-type)-TMPRSS2 interactions for different active sites of ACE2 and the interaction of mutated ACE2-TMPRSS2 obtained from docking simulation have been represented. In all mutated ACE2-TMPRSS2 docking simulations, Arg652 was considered as the active residue of ACE2. Letters B and C indicate ACE2 and TMPRSS2 chain IDs, respectively. The green line represents the hydrogen bonds. Residues involved in hydrogen bonds have been shown in blue and green colors, while brown and black colors have been used for hydrophobic interactions. The length of the hydrogen bonds is expressed in terms of Angstrom. **a)** TMPRSS2/ACE2(Wild-type), Arg 710 as the active residue of ACE2 in the docking simulation; **b)** TMPRSS2/ACE2(Wild-type),Arg708 as the active residue of ACE2 in the docking simulation; **c)** TMPRSS2/ACE2(Wild-type), Arg652 as the active residue of ACE2 in the docking simulation; **d)** TMPRSS2-Mutated ACE2(ALA650>SER) **e)** TMPRSS2-Mutated ACE2(ARG708>GLN) **f)** TMPRSS2-Mutated ACE2(ARG708>TRP)

**
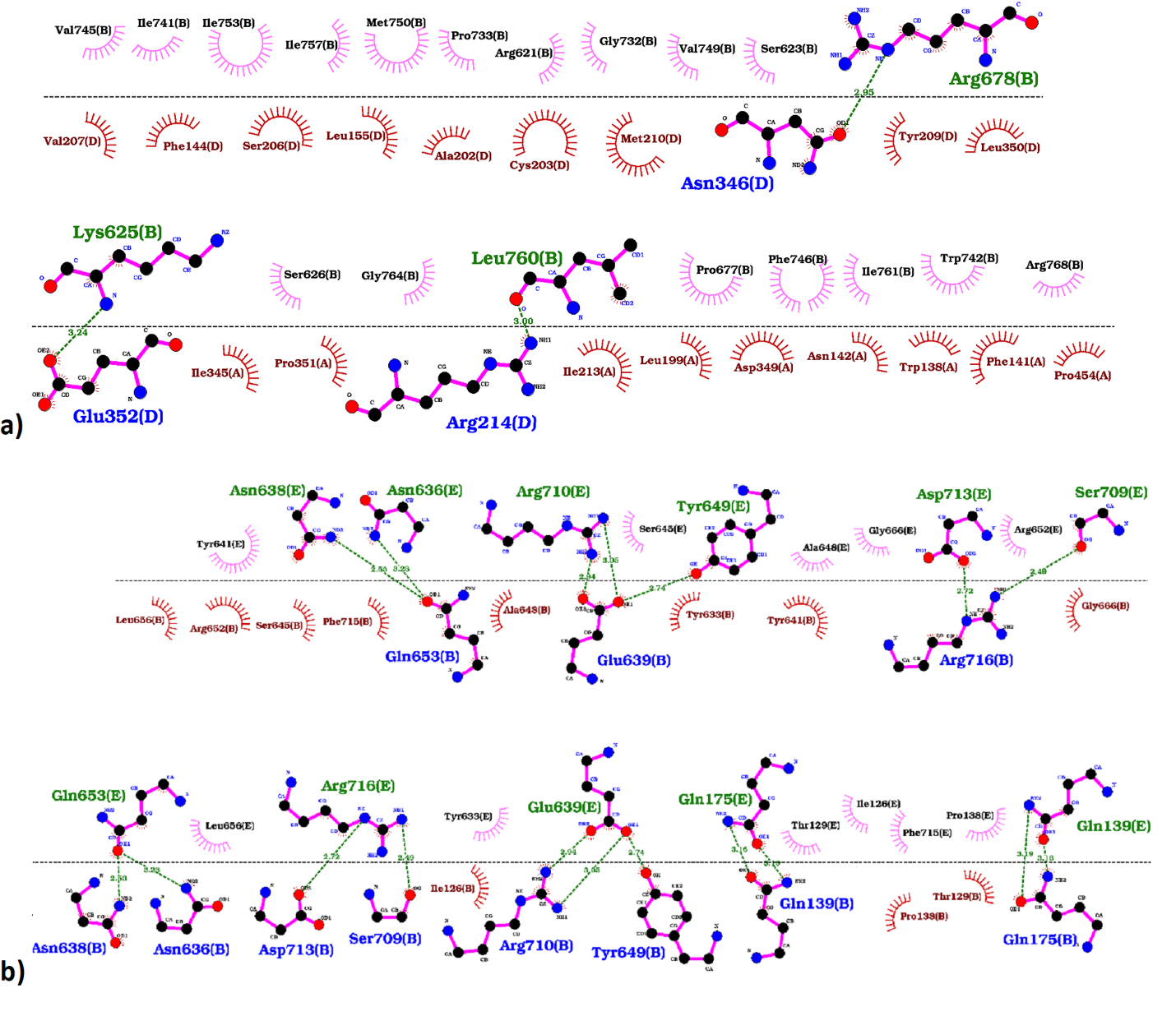
**

**Fig. S2: Result of analyzing the interaction of ACE2-B(0)AT1 and ACE2 dimer using LigPlus Software**

Letter B and D indicate ACE2 and B(0)AT1 chain IDs respectively. In the dimer of ACE2, the second chain has been labeled by letter E. The green line represents the hydrogen bonds. Residues involved in hydrogen bonds have been shown in blue and green colors, while brown and black colors are used for hydrophobic interactions. The length of the hydrogen bonds is expressed in terms of Angstrom.

**a) ACE2 receptor interacts with protein B(0)AT1 or SLC6A19 interactions**

The ACE2 and B(0)AT1 complex obtained from 6M17 PDB file. These molecules are chain B and A in the 6M17 file.

**b) Analysis of ACE2 receptor dimerization**

The ACE2 dimerization was obtained from the 6M17 PDB file. These molecules are chain B and D in the 6M17 file.


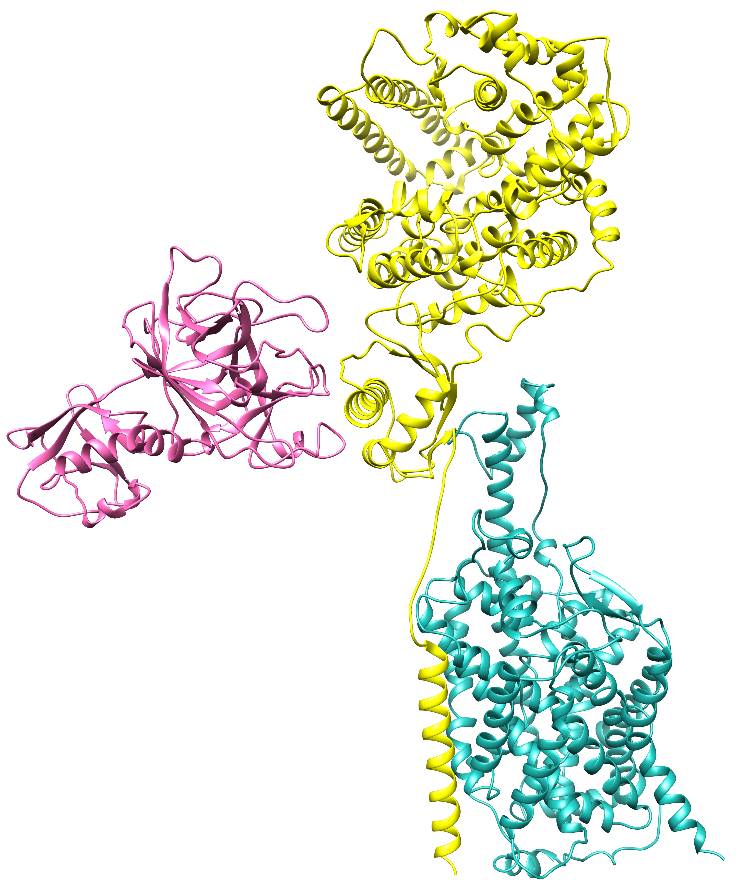


a)


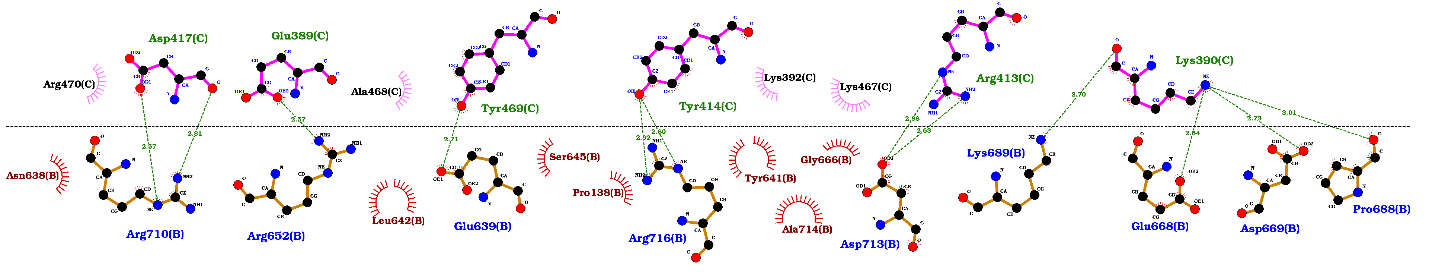


b)

**Fig. S3: Result of the docking simulation of ACE2-B(0)AT1 complex and TMPRSS2**

a) The conformation of B(0)AT1-ACE2-TMPRSS2 complex obtained from docking simulation of the ACE2(yellow)-B(0)AT1(light sea green) and TMPRSS2 (hot pink). The ACE2-B(0)AT1 has been extracted from the experimental 3D structure (6M17 PDB file). There is not any spatial restriction or conflict between ACE2 partners (B(0)AT1 and TMPRSS2). The Haddock score is -111+-1.3 and z-score =-1.4. This figure has been produced by the UCSF Chimera 1.14-linux_x86_64 (<https://www.cgl.ucsf.edu/chimera/download.html>). b) The details of interactions between TMPRSS2 and the ACE2-B(0)AT1 complex obtained from docking simulation have been represented. Letters B, C, and D indicate ACE2, TMPRSS2, and B(0)AT1 chain IDs, respectively. The green line represents the hydrogen bonds. Residues involved in hydrogen bonds have been shown in blue and green colors, while brown and black colors have been used for hydrophobic interactions. There is not any residue from chain D in the interactions thus there is not any interaction between TMPRSS2 and the B(0)AT1. The length of the hydrogen bonds is expressed in terms of Angstrom.
